# The Story of the Insane from Year to Year

**Published:** 1903-08-01

**Authors:** 


### THE STORY OF THE INSANE FROM YEAR TO YEAR.

Fifteenth Annual Series.

### GLASGOW DISTRICT ASYLUM AT WOODILEE.

At this asylum an increase during the year from 847 to 881 has to be noted. There were 239 first admissions, 64, readmissions, 116 recoveries, 77 discharged not recovered, and 76 died. The daily average number resident was 854, and the total number under treatment was 1,150. Calculated on the admissions the recoveries stand at 38·28 per cent., and calculated on the average number resident the deaths stand at 8 8 per cent. Of the year's deaths 36 were caused by cerebral and spinal diseases, 9 by consumption, and 4 by other lung diseases. Of the year's admissions only 6 seem to have had the insanity caused by moral causes, and of the physical causes drink heads the list with 62 cases, previous attacks account for 47, syphilis for 28, and heredity for 19. The number of readmissions to our public asylums has led some people to believe that insanity is not often really recovered from; and Dr. Marr tells us that this belief is held not only by intelligent laymen but by some medical men also. This belief, he adds, is "no more applicable to insanity than it is to other bodily disorders, save in the light that once a person has had an attack of insanity he is thereby predisposed more readily to a second attack." The truth is that patients discharged recovered from our asylums very often revert to their evil ways of living and speedily become again insane; but had they conducted themselves in a proper manner the second attack might never have occurred. In this asylum over 20 per cent. of the admissions were traced to alcoholic excess, and Dr. Marr points out that epilepsy is often caused by intemperance, and certainly epilepsy often causes insanity, so that the direct and indirect effects of alcohol in the production of this disease would amount to a very high total. Woodilee has always had the reputation of being one of the most advanced asylums in Great Britain. It is clear that it intends to keep its place in the front rank, for it is now adding a reception house and a sanatorium for consumption. The former is to be used as an admission ward; and it is intended that all new patients should be kept therein until their condition has been carefully investigated. The sanatorium is composed of three separate blocks and an administrative department. It will contain 62 beds. When speaking of the training of attendants and nurses, Dr. Marr states that the teaching staff has considered it advisable to go beyond the training required for the medico-psychological certificate, and institute a three or four years' course of instruction, after which the attendants and nurses will submit to a more searching examination, and will obtain a certificate signed by the whole of the teaching staff of the asylum. For many years we have urged the adoption of this step by all the public asylums of the country; and we are not a little pleased to be able to record that Woodilee has joined the little band of pioneers in this great work. The committee say that Dr. Marr has given evidence of his intention that the administration of the asylum and the treatment of the insane shall be maintained in the highest efficiency. The weekly rate of maintenance is 9s. 6d. per head.

